# Occurrence of multiclass endocrine disrupting compounds in a drinking water supply system and associated risks

**DOI:** 10.1038/s41598-020-74061-5

**Published:** 2020-10-20

**Authors:** Sze Yee Wee, Ahmad Zaharin Aris, Fatimah Md. Yusoff, Sarva Mangala Praveena

**Affiliations:** 1 Department of Environment, Faculty of Forestry and Environment, Universiti Putra Malaysia, 43400 Serdang, Selangor Malaysia; 2 Department of Aquaculture, Faculty of Agriculture, Universiti Putra Malaysia, 43400 Serdang, Selangor Malaysia; 3 Department of Environmental and Occupational Health, Faculty of Medicine and Health Sciences, Universiti Putra Malaysia, 43400 Serdang, Selangor Malaysia; 4 International Institute of Aquaculture and Aquatic Sciences, Universiti Putra Malaysia, 71050 Port Dickson, Negeri Sembilan Malaysia

**Keywords:** Environmental chemistry, Environmental impact, Environmental chemistry

## Abstract

Contamination by endocrine disrupting compounds (EDCs) concerns the security and sustainability of a drinking water supply system and human exposure via water consumption. This study analyzed the selected EDCs in source (river water, *n* = 10) and supply (tap water, *n* = 155) points and the associated risks. A total of 14 multiclass EDCs was detected in the drinking water supply system in Malaysia. Triclosan (an antimicrobial agent) and 4-octylphenol (a plasticizer) were only detected in the tap water (up to 9.74 and 0.44 ng/L, respectively). Meanwhile, chloramphenicol and 4-nonylphenol in the system were below the method detection limits. Bisphenol A was observed to be highest in tap water at 66.40 ng/L (detection: 100%; median concentration: 0.28 ng/L). There was a significant difference in triclosan contamination between the river and tap water (*p* < 0.001). Overall, the life groups were estimated at no possible risk of EDCs (risk quotient < 1). Nonetheless, the results concern the transport and impact of EDCs on the drinking water supply system regarding treatment sustainability and water security. Further exploration of smart monitoring and management using Big Data and Internet of Things and the need to invent rapid, robust, sensitive, and efficient sensors is warranted.

## Introduction

Endocrine disrupting compounds (EDCs) have emerged as contaminants warranting concern recently because of their wide application and elevated contamination in various environmental compartments^1,2^. These environmental contaminants can be classified based on their potential endocrine disruption effects (e.g., carcinogenic, genotoxic, cytotoxic, and neurotoxic), impacting individual and population growth and development as well as socioeconomic values. However, there is an insufficient national and international level of concern and practice despite ongoing scientific efforts to reveal the nature, extent, and risks associated with the broad scope of compounds; meanwhile, the relatively low level of public awareness requires addressing^3^.

A drinking water supply system generally consists of a drinking water source (the raw water to be treated), drinking water treatment plant, and drinking water supply (the distributed treated water). Surface river water is the main source of the raw water intake performed by drinking water treatment plant for treatment and subsequent supply of drinking water in most countries. Point sources (e.g., treated and nontreated discharges) and nonpoint sources (e.g., runoff and leachate) load EDCs into the raw water body^4–8^. The drinking water treatment plant is the last point of protection from chemical exposure between the environment (i.e., drinking water source) and the users. Unfortunately, incomplete removal of EDCs by conventional treatments occurs since the treatment process has not been designed for EDC elimination, contributing to EDC loading in the global drinking water supply i.e., tap water^8–12^.

Previous studies have reported on the higher efficiency of advanced remediation methods—for example, ozonation, granular activated carbon, and powdered activated carbon—in EDC removal, with only trace concentration of EDCs found in treated water. Nonetheless, advanced treatments that incorporate a multibarrier system (efficiency differs widely in accordance with several variables) are challenging to deploy so as to upgrade the removal efficiency of conventional drinking water treatment plants. The cost-effectiveness and process sustainability are also of concern.

EDC loading in drinking water supply systems, from the source to the supply, provokes issues in terms of the security and sustainability of the system. This is in regard to not only to the pollution level but also organism exposure to EDCs, especially humans, via drinking water consumption. EDCs are commonly known to affect the endocrine system, impacting environmental sustainability, public health (triggering both acute and chronic diseases), and economic well-being^3^.

Thus, the present study conducted in Malaysia aims to reveal the selected EDCs along the route from source to supply to quantify the possible impact on the drinking water supply system in terms of treatment sustainability, water security, and health risk issues. The human health implications due to the ingestion of multiresidual EDCs via drinking water in children and adults are also ascertained based on the risk quotient (RQ) method. New datasets on the level of magnitude and profile of EDC pollution in drinking water supply systems can be adopted by related stakeholders, authorities, and industries to drive decision-making and planning efforts concerning water monitoring and management. The present study closely links the aspects of water security and sustainability to the following Malaysian concepts or initiatives:

- The National Water Resources Policy, which aims “to provide clear directions and strategies for water resources management, including collaborative governance to ensure water security and continued sustainability”
- National Key Results Areas, which concern efforts “to improve basic infrastructure, ensuring public access to clean and treated water”
- The RMK-11 11th Malaysia Plan, which “will ensure sustainability of the nation’s natural resources; minimize pollution; and strengthen energy, food, and water security”

Moreover, an overview of the occurrence and risk of emerging EDCs from source to supply provides insights into related aspects of environmental sustainability (pollution control, resource utilization, and best practices), economic prosperity (operational efficiency, capital budgeting, and investment appraisals), and public health protection (water security, disease control, and regulated human exposure). Water monitoring and management practices in regard to environmental EDCs to ensure safe water resources, public health, and economic prosperity are parallel to the following Sustainable Development Goals (SDGs): Goal 6, Clean water and sanitation; Goal 3, Good health and well-being; and Goal 8, Decent work and economic growth.

## Results and discussion

### Occurrence and comparison of the target multiclass EDCs in the drinking water supply system

**Figure 1:**
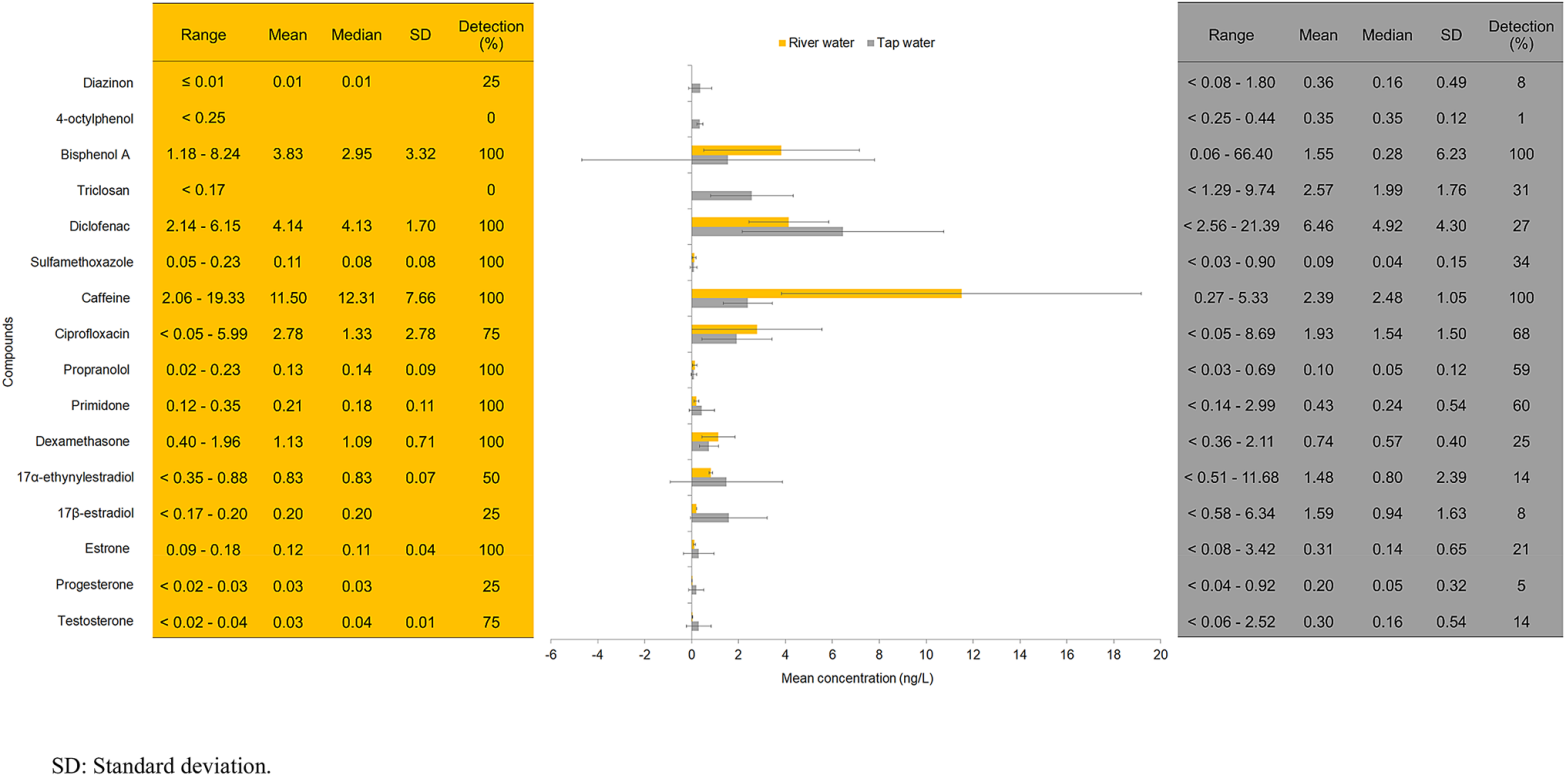
Comparison of the target multiclass EDCs in the drinking water supply system.

A mixture of EDCs (14 compounds) was present in the Malaysian drinking water supply system under assessment, including both at the drinking water source (river water) and supply (tap water) points. The EDCs included (1) hormones, i.e., testosterone, progesterone, estrone, 17β-estradiol, and 17α-ethynylestradiol; (2) pharmaceuticals, i.e., dexamethasone, primidone, propranolol, ciprofloxacin, caffeine, sulfamethoxazole, and diclofenac; (3) plasticizer, i.e., bisphenol A; and (4) pesticide, i.e., diazinon. Chloramphenicol and 4-nonylphenol remained below method detection limits in both the river and tap water samples. Triclosan and 4-octylphenol were only detected in tap water at concentrations of up to 9.74 and 0.44 ng/L, respectively. Figure 1 compares the target multiclass EDCs in the drinking water supply system. Of the 16 EDCs, only caffeine and bisphenol A were detected in all tap water samples (100% detection), with the level of bisphenol A reaching up to 66.40 ng/L (range 0.06–66.4 ng/L; median concentration: 0.28 ng/L).

Bisphenol A and triclosan are correlated with an increased risk of male subfertility due to altered hormone levels following short- or intermediate-term exposure^13^. Particularly, triclosan impacts the functioning of Sertoli and Leydig cells, leading to reduced sperm production and elevated luteinizing hormone levels, respectively, whereas bisphenol A is negatively associated with the testosterone level in the human body. Following their investigation, Wu et al*.*^14^ reported on the impact of bisphenol A and triclosan in changing the thyroid hormone level (i.e., inhibited iodide uptake and manipulated thyroid hormone synthesis-related gene expression and the thyroid peroxidase activity), with resultant possible growth and mental disruption, metabolic destruction, and brain damage and an increased mortality rate. Furthermore, bisphenol A displays potential cardiotoxic and embryotoxic impacts with reduced cell viability and myocardial differentiation as well as combined effects (synergistic and additive) under multiresidue interaction^15^. Also, bisphenol A and triclosan exposure (acute and chronic) caused metabolic alterations, i.e., increased lipid accumulation and impaired lipid metabolism (e.g., disrupted lipid synthesis, transport, and degradation) in zebrafish (*Danio rerio*); moreover, triclosan triggered most severe hepatic steatosis^16^. Ecological and human exposure to bisphenol A are of great concern especially given bisphenol A showed the third-highest mean concentration in both the river water (before treatment) and tap water (after treatment), following caffeine and diclofenac, which ranked first and second, respectively (Fig. 1).

The highest mean concentration of caffeine, an anthropogenic marker, found in the drinking water supply system may cause neurological illness (due to inhibiting acetylcholinesterase activity), oxidative stress (due to increasing 7-ethoxyresorufin-*O*-deethylase, glutathione S-transferase, and superoxide dismutase), and feminization (due to inducing vitellogenin in serum) as tested in goldfish (*Carassius auratus*)^17^. Additive and antagonistic effects have been observed under mixture exposure with sulfamethoxazole, an antibiotic that was also present in the drinking water supply system at mean concentrations of 0.11 and 0.03 ng/L in river and tap water, respectively. Similarly, diclofenac exposure (as low as 29.6 ng/L in 8 days) may lead to sexual differentiation based on estrogenic effects and alteration of gene expression (gonadotropin and vitellogenin), interrupting the hypothalamus–pituitary–gonad axis (leading to an imbalance in sex steroid levels with reduced androgen/estrogen ratio) and affecting mating vocalizations (i.e., resulting in impaired calling behaviors with lowered mating and reproduction rates)^18,19^. Hormones, i.e., testosterone, progesterone, estrone, 17β-estradiol, and 17α-ethynylestradiol, were observed at mean concentrations ranged from 0.03 to 0.83 ng/L and 0.20 to 1.59 ng/L in river and tap water, respectively. However, the trace levels of hormones should not be neglected—for example, 17α-ethynylestradiol has the potential to trigger various endocrine dysfunction effects at exposure levels as low as 1 ng/L^4^.

Meanwhile, it is of great concern that diclofenac was observed in tap water at a median concentration of 4.92 ng/L (range < 2.56–21.39 ng/L), in comparison with other EDCs detected at median concentrations below 2.50 ng/L (Fig. 1). Moreover, the contamination level was higher relative to that in the drinking water supply in Portugal (not detected), Japan (range < 2.50–16.00 ng/L; median concentration < 2.50 ng/L), and the United States (range 0–9.40 ng/L; median concentration: 0 ng/L)^6,8,12^. Notably, advanced processes ensure relatively greater removal efficiency as compared with conventional processes. Meanwhile, median concentrations of the multiclass EDCs in the river water were mostly in the range of 0.01–1.33 ng/L, with the exceptions of bisphenol A, diclofenac, and caffeine (2.95, 4.13, and 12.31 ng/L, respectively). The median concentration of sulfamethoxazole (0.08 ng/L) in the drinking water source in the present study was comparatively lower than those of other, developed countries (3.20–16.00 ng/L); nonetheless, it was not detected in the drinking water supply after treatment in the developed countries, where advance processes were able to remove nearly all traces completely^8^.

Out of the 14 EDCs, estrone, dexamethasone, primidone, propranolol, caffeine, sulfamethoxazole, diclofenac, and bisphenol A were observed in all river water samples collected (100% detection). The observed contamination by these EDCs was the result of anthropogenic sources due to manufacturing and the divergent usage of a broad scope of EDCs-containing products^2,20^. Because of their high persistence and resistance to transformation (chemical, physical, and biological), EDCs persist and disperse in relation to (1) industrial and municipal discharges, (2) treatment processes, and (3) environmental processes such as runoff and infiltration^4–8^. Both conventional and advanced technologies in wastewater and sewage treatment have also not been specifically designed to remediate EDCs and are therefore overcome by the characteristics of EDCs such as persistence and hydrophilicity^2^.

Variations in the occurrence and distribution of EDCs in household tap water from the same drinking water treatment plant were potentially the result of their dynamics and partitioning in the distribution network prior to reaching consumers’ taps. The design and operation of the water distribution system are varied in terms of pumping, piping, storage, water use patterns, and other hydraulic factors as a result of several influences such as housing type and construction phase. These factors can cause spatial variations in physical and chemical properties (e.g., contamination levels of heavy metals, organic matter, microorganisms, and disinfection by-products) in tap water^21^. Throughout its development phases, Malaysia has used various types of pipe materials such as mild steel, asbestos cement, unplasticized polyvinyl chloride, ductile iron, and high-density polyethylene; meanwhile, eventual replacement of aging asbestos cement with ductile iron and high-density polyethylene to cope with leaking and bursting due to changes in pressure and weather has occurred^22^. The condition of pipes (e.g., corrosion, leaching, and leaking) in the water distribution system also affects the residence of contaminants in the drinking water supply^23^. Future studies on the factors influencing the dynamics, loading, and partitioning of EDCs in the system are of great significance to support scientific developments and the attainment of technical solutions for ensuring good provision and quality of the drinking water supply.

The independent *t* test revealed a significant difference existed in triclosan contamination between river and tap water [*t*(153) = 6.362; *p* < 0.001]. Meanwhile, turbidity (519.7 ± 410.6 NTU) was statistically significantly reduced in tap water (0.9 ± 0.3 NTU) after the treatment process [*t*(4) =  − 2.285; *p* < 0.05]. Significant variations were discovered in the pH level between river (6.98 ± 0.25) and tap water (7.55 ± 0.34) [(157) = 3.724; *p* < 0.001]; however, the levels remained within the guidelines set forth by the Malaysia Drinking Water Quality Standard (6.5–9.0) as well as the Australian Drinking Water Guidelines, World Health Organization (WHO) Guidelines for Drinking Water Quality, and United States Environmental Protection Agency Drinking Water Standards (acceptable range 6.5–8.5). Moreover, the regulated water quality parameters were in accordance with the standards implemented to control such. Conversely, the presence of EDCs in the drinking water supply system indicates the need for the addition of further scientific developments and technical solutions to the existing monitoring and management framework with regard to emerging contaminants, especially to support legislative and policy ratification. The WHO highlighted previously that the Guidelines for Drinking Water Quality are inadequate for regulating EDCs in drinking water^24^. Notably, there is an absence of considerations of most emerging contaminants in existing drinking water regulatory compliances^3^.

As depicted in Fig. 1, treatment efficiency and sustainability are the key matters of concern when EDC levels are higher in treated water than raw water. The higher concentrations of testosterone, progesterone, estrone, 17β-estradiol, 17α-ethynylestradiol, primidone, diclofenac, triclosan, 4-octylphenol, and diazinon in tap water relative to those in river water may be potentially attributed to (1) EDC desorption during operation, (2) the presence of saturated absorbents with lower adsorption rates, (3) dissolution of EDC aggregates, and/or (4) the formation of parent compounds from metabolites^25,26^. Moreover, drinking water sources with a high content of EDCs (due to insufficient wastewater and sewage treatment with discharge of EDC-containing effluents to the influents of drinking water treatment plants) also impacted the efficiency of EDC removal in the drinking water supply^8,27^. Finally, contamination in the pipes, especially those treated by epoxy coating, contributed to the leaching of plasticizers into the drinking water supply^23^.

### Human health risk

The level of exposure to EDCs via drinking water intake was based on drinking water ingestion according to body weight. Among a total of 257 participants, the age groups included children (*n* = 118, aged < 20 years) and adults (*n* = 139, aged ≥ 20 years), which were classified based on research by Amarra et al*.*^28^ and Bujang et al*.*^29^. Based on the questionnaire survey, body weight and daily water intake of children (25.30 kg; 1.26 L/day) and adults (68.39 kg; 1.86 L/day) were employed for human health life-stage risk estimation based on the conservative worst-case scenario, where the risks of continuous EDC exposure via drinking water intake on a regular basis exist. Consumers’ frequency of exposure was 1 (365 days/365 days) (95th-percentile value). The acceptable daily intake (ADI) of the observed EDCs ranged from 15 to 150,000 ng/kg/day^6,30–35^. Remarkably, the estimated daily intakes (EDIs) of the observed EDCs for the life groups in the present study were less than the corresponding ADIs (Table 1).

**Table 1:** EDIs of detected EDCs for children and adults in river and tap water.

EDCs	River water			Tap water		
	C_s_ (ng/L)	EDI via drinking water (ng/kg/day)		C_s_ (ng/L)	EDI via drinking water (ng/kg/day)	
		Children	Adults		Children	Adults
**Hormone**						
Testosterone	0.04	0.003	0.001	2.52	0.162	0.071
Progesterone	0.03	0.002	0.001	0.92	0.059	0.026
Estrone	0.18	0.012	0.005	3.42	0.219	0.096
17β-Estradiol	0.20	0.013	0.006	6.34	0.406	0.178
17α-Ethynylestradiol	0.88	0.056	0.025	11.68	0.748	0.327
**Pharmaceutical**						
Dexamethasone	1.96	0.125	0.055	2.11	0.135	0.059
Primidone	0.35	0.022	0.010	2.99	0.191	0.084
Propranolol	0.23	0.015	0.006	0.69	0.044	0.019
Ciprofloxacin	5.99	0.383	0.168	8.69	0.556	0.243
Caffeine	19.33	1.237	0.541	5.33	0.341	0.149
Sulfamethoxazole	0.23	0.015	0.006	0.90	0.058	0.025
Diclofenac	6.15	0.394	0.172	21.39	1.369	0.599
Triclosan	NA	NA	NA	9.74	0.624	0.273
**Plasticizer**						
Bisphenol A	8.24	0.527	0.231	66.40	4.249	1.859
4-octylphenol	NA	NA	NA	0.44	0.028	0.012
**Pesticide**						
Diazinon	0.01	0.001	< 0.001	1.80	0.115	0.050

*C*_*s*_ maximum detected concentration, *EDI* estimated daily intake, *NA* not available.

**Figure 2:**
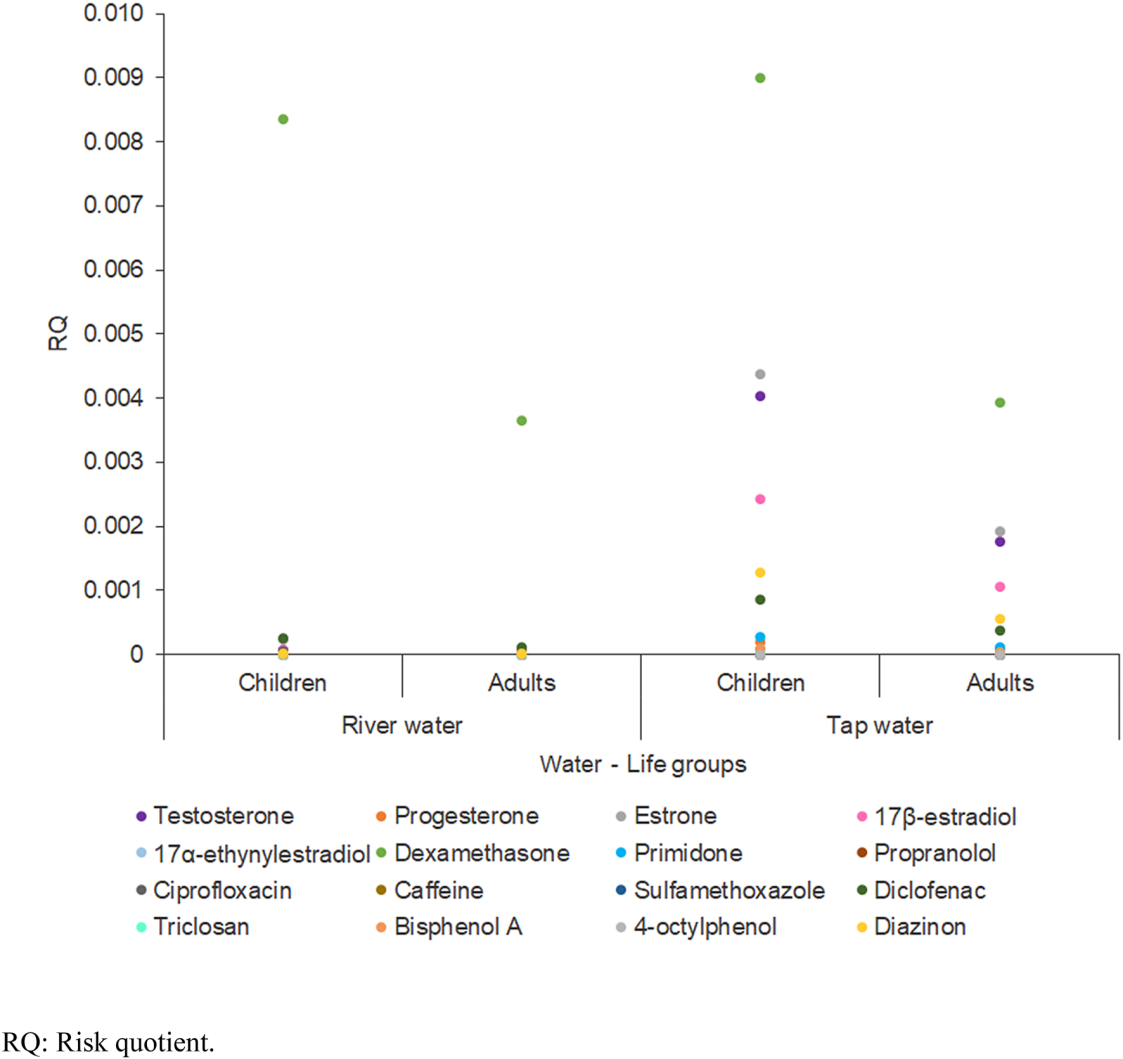
RQ profiles of detected EDCs in river and tap water with respect to life groups via daily water intake.

All the EDCs tested showed no potential risk (RQ < 1) to humans during daily water intake (Fig. 2). Bisphenol A was detected at the highest level at 66.40 ng/L; thus, the daily intake of bisphenol A via tap water consumption was the highest among all EDCs throughout the life groups (children: 1.86 ng/kg/day; adults: 4.25 ng/kg/day). However, dexamethasone, which was detected at a level of 2.11 ng/L, possessed the highest risk to humans (RQ_children_ = 0.009; RQ_adults_ = 0.004) via tap water consumption in the present study because of its lower ADI, i.e., 15 ng/kg/day (Fig. 2). Dexamethasone exposure is associated with reproductive impairment, which is caused by oxidative stress and lowered steroid hormone levels^36^.

**Figure 3:**
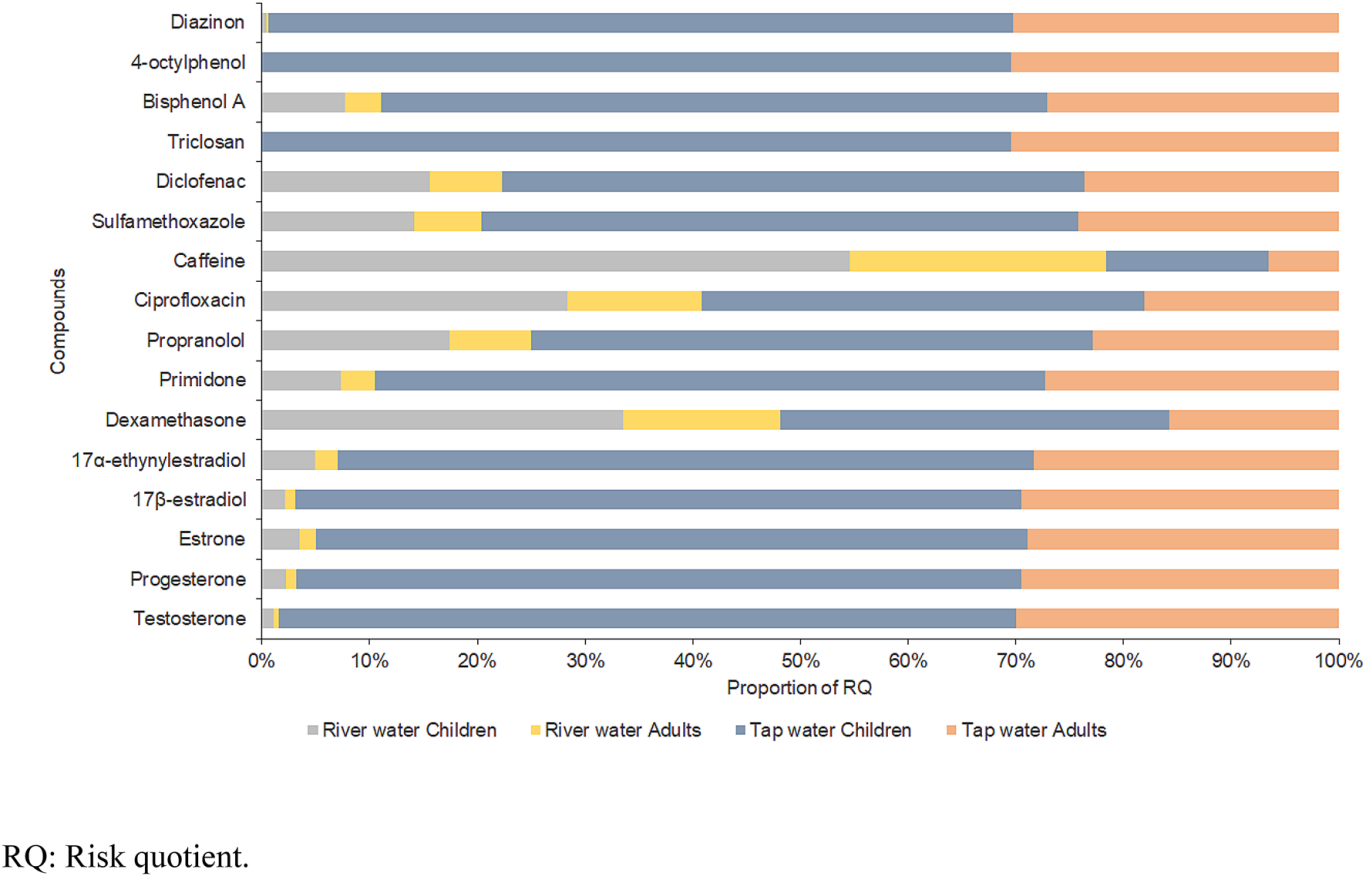
Proportion of RQ values of detected EDCs in river and tap water with respect to life groups via daily water intake.

Tap water (RQ ≤ 0.009) possessed a higher risk than river water (RQ ≤ 0.008) in the context of EDC exposure, except with respect to caffeine (Fig. 3). The RQ of caffeine in river water (RQ = 0.000008) was at least four times greater than that in tap water (RQ = 0.000002). The present comparison of EDCs in the drinking water supply system (between river and tap water) depicted the difference in the exposure levels and potential risks of EDCs from two different sources. Also, the human health risk of EDCs in the untreated raw water suggests consumers, especially those in rural areas and developing countries, are still having problems with accessing potable water and are using untreated raw water (e.g., river water and groundwater) as their daily drinking water supply. In one research, the drinking water extracted from shallow wells in Kenya contained sulfamethoxazole (up to 30 ng/L)^37^. Figure 3 demonstrated the proportion of RQ values of detected EDCs in river and tap water among life groups due to daily water intake. Children showed a higher risk of EDC consumption relative to adults (RQ was 2.3 times greater). The accounted higher exposures and thus greater risks earlier in life could be attributed to the greater drinking water intake on a body-weight basis demonstrated by children. Further, the difference may also be attributed in part to varying physiological characteristics and everyday activities between the life groups^38^.

Apparently, the conventional treatment method—particularly, aeration, coagulation, and flocculation, sedimentation/clarification, filtration, disinfection, and pH adjustment—used presently in drinking water treatment plants in Malaysia is inadequate to eliminate contamination and thus impacts the quality of the drinking water supply. Reductions in the concentrations of steroid hormones and plasticizers (approximately 95–100%) by means of common disinfection means such as chlorination and chloramination suggests the efficiency of using disinfectants in the drinking water distribution system^39–42^. However, this process was observed in correlation with an increase in the generation of potential carcinogenic disinfection by-products, including trihalomethanes and halogenated acetic acids. Meanwhile, during the pharmaceutical removal process, various disinfection processes have been linked to transformation of the compounds, potentially producing by-products that are more toxic than the parent compounds^43^. With continuous consumption and long-term exposure, the subsequent potential human health effects of such an EDC mixture (i.e., parent compounds, metabolites, and other pollutants) are of significant concern even though they exist at trace levels and the precise mechanism has yet to be determined.

### Smart drinking water supply system monitoring and management

Despite the negative impacts of the Industrial Revolution on environmental pollution and health, the resultant developments and increased sophistication of technology may also contribute to political, economic, social, and environmental improvements. The Internet of Things (IoT) is one of these inventions, which is connected with almost every industry ranging from smart home to smart city. Remarkably, IoT has been widely utilized in environmental fields to deal with the need for continuous monitoring, management, control, prediction, and logistics as well as to ease planning and decision-making among citizens and authorities^44^. Further, its applications in the water industry include the sorting of water scarcity by sensing chemical contents (e.g., chlorine) and physicochemical properties (e.g., pH, temperature, conductivity, and turbidity) that can impact the natural state of water. Within the communication arrangement, a wireless sensor network (WSN), which combines sensing devices and the monitoring of multiple water quality parameters, has been widely utilized, as noted in previous studies^45–47^. The implementation of IoT technology in EDC monitoring requires a breakthrough since the broad scope of EDCs in interference-containing environmental matrices at trace levels necessitate the involvement of analytical protocols within a vast quality assurance and control structure. Further, the characteristics and mechanisms of emerging contaminants remain largely unknown. Several sensors such as electrochemical sensors, biosensors, and immunosensors have been widely used for environmental applications; however, they still show some limitations such as single analyte determination, low biological material stability, application in selective matrices, and relatively high detection limits^48^. Thus, science–industry collaborations aimed at inventing rapid, robust, sensitive, and efficient sensors for the detection and quantification of the environmental EDCs that commonly exist at trace levels is of interest.

**Figure 4:**
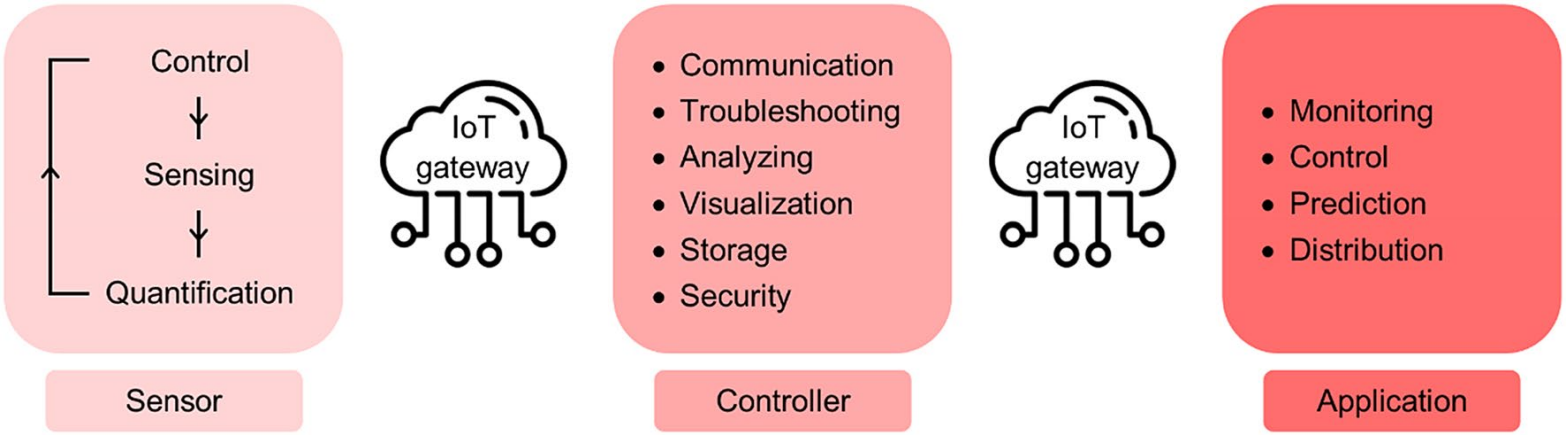
Integration of Big Data and IoT in monitoring and managing EDCs in the drinking water supply system. Icon courtesy of Freepik (https://www.freepik.com).

The integration of Big Data and IoT in monitoring and managing EDCs in the drinking water supply system is expected to be essential in data collection, processing, and transfer, among sensors–controllers-applications (Fig. 4). With the integration of Big Data and IoT in measuring, diagnosing, and sorting information from devices, the data can be further transferred to a central information hub for immediate communication, troubleshooting, analyzing, visualization, storage, and security. Subsequently, all information should be made available and disseminated to the stakeholders, authorities, and industries, especially the public, in the interest of community-based risk governance and communication, which can be expected to be essential in supporting sustainable development, monitoring, and management through risk behavior development and participation^3^. This technology, which is known as a power-efficient and simpler solution, represents a smart tool in the monitoring and management of emerging contaminants and their associated risks. Subsequently, the smart application facilitates a multibarrier approach in the area of drinking water supply system monitoring and management to assure safe water resources^2^.

## Conclusion

Among 18 screened compounds, 14 multiclass EDCs were detected in the drinking water supply system, encompassing both drinking water source (river water) and supply (tap water). Chloramphenicol and 4-nonylphenol were below the method detection limits in both the drinking water source and supply samples. Tap water also contained triclosan and 4-octylphenol at concentrations of up to 9.74 ng/L and 0.44 ng/L, respectively. The highest level among the EDCs detected was 66.40 ng/L (bisphenol A) in Malaysian tap water. Emerging contamination from source to supply concerns the operation of the drinking water supply system with respect to security and sustainability. The issues of (1) polluted drinking water source, (2) insufficient removal efficiency of treatment practices, (3) human exposure through daily water ingestion, and (4) potential risk of EDC exposure at different life stages of consumers are matters of concern. Moreover, the drinking water supply system was observed to show higher concentrations of testosterone, progesterone, estrone, 17β-estradiol, 17α-ethynylestradiol, primidone, diclofenac, triclosan, 4-octylphenol, and diazinon in tap water. Generally, the risk of EDCs was higher in tap water (RQ ≤ 0.009) as compared with in river water (RQ ≤ 0.008) in the present study, except with regard to caffeine. Children were exposed to a greater risk of EDCs compared to adults (RQ_children_ > RQ_adults_) when considering body weight. Overall, however, no possible health risk of EDCs (RQ < 1) was estimated to exist in the different life groups as a result of consuming drinking water. In the context of the monitoring and management of environmental EDCs, regulating adverse impacts, bridging the knowledge gap, and smart applications are required to support a multibarrier approach in the regulation of the drinking water supply system.

## Methods

### Sampling and sample analysis

**Figure 5:**
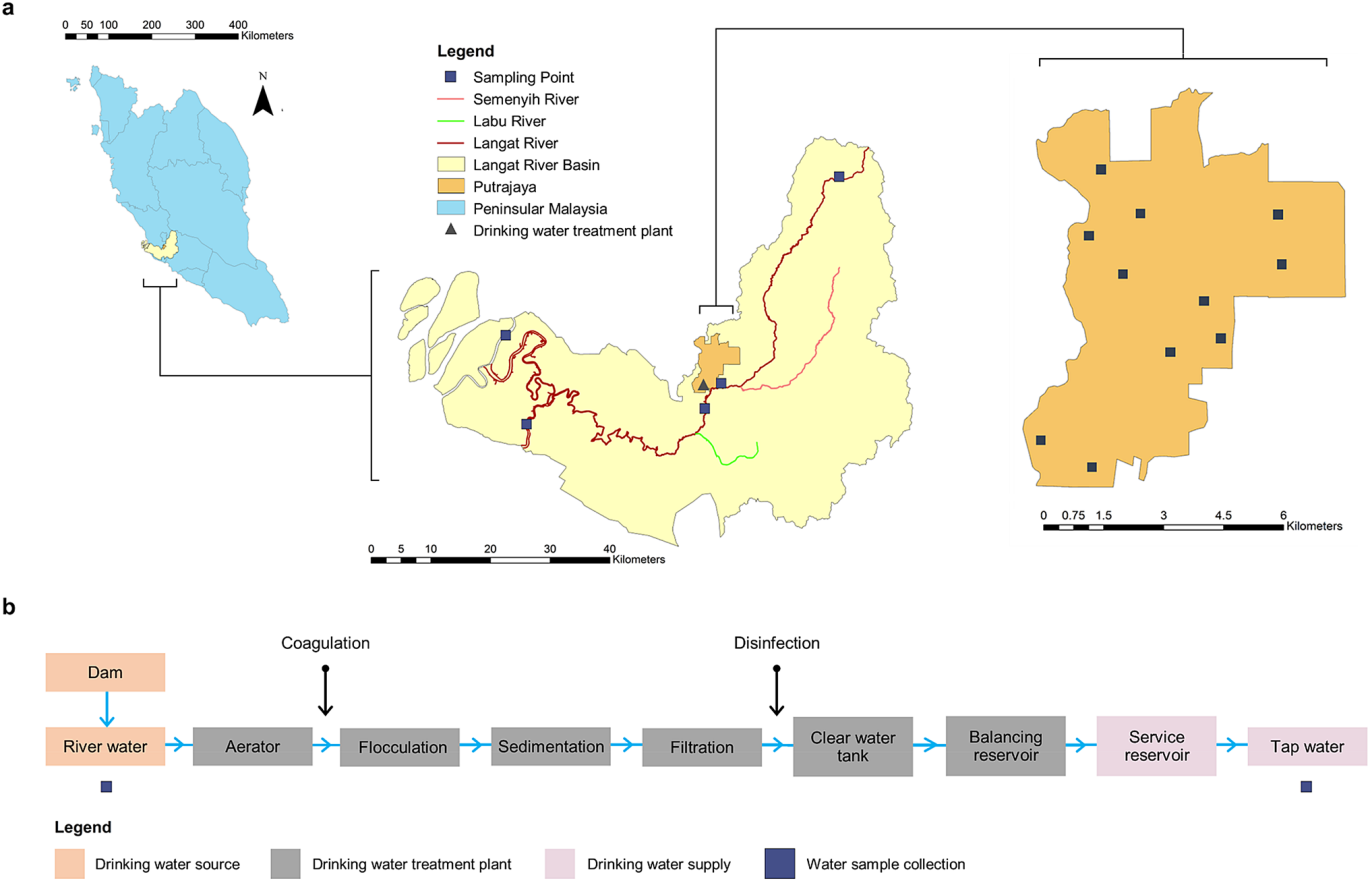
(**a**) Map of the study area and sampling points in the present study and (**b**) flow diagram of the water treatment process and water sample collection in the drinking water supply system. Maps are generated using ArcGIS (Version 10.4.1, https://desktop.arcgis.com/en/arcmap/), and then organized and labeled in Microsoft Publisher (Version 2016, https://www.microsoft.com/en-my/microsoft-365/publisher) [Software].

The Greater Kuala Lumpur, or the Klang Valley (GKL/KV), is a National Key Economic Area with extensive urbanization and a population of approximately 7.2 million that is predicted to increase by 39% to 10 million in 2020 and about 20 million by 2030^49^. Subsequently, the water demand is projected to double from 3900 to 7800 million L/day by 2034. Putrajaya acts as the federal administrative center of Malaysia, located in GKL/KV. It is a planned city under different phases of construction begun in 1996. By now, it has an estimated household number of 19,511 with approximately 88,300 people. Figure 5a depicts the map of the study area and sampling points in the present study. The drinking water supply in Putrajaya is sourced and treated from the Langat River (approximately 141 km long) for consumers’ consumption. Notably, the Langat River is exposed to diverse pollution sources such as domestic discharges, agro-based industries/farming discharges, runoffs from earthworks and land clearing, and the effluents of manufacturing activities^50^. As depicted in Fig. 5b, drinking water treatment plant employs conventional treatment methods, including aeration, coagulation and flocculation, sedimentation/clarification, filtration, disinfection, and pH adjustment^51^.

Water sampling in the present study involved collection from both drinking water source and drinking water supply points (river and tap water, respectively). A questionnaire survey of consumers’ characteristics such as body weight, daily drinking water intake, and frequency of exposure for human health risk assessment was deployed. Based on research by Daniel^52^, Lichtenberg^53^, Prüss-Ustün et al*.*^54^, and Suresh and Chandrashekara^55^, the necessary representative sample size for this study was calculated at a minimum of 140 households after taking in all the relevant considerations for determining the sample size and was coupled with an extra 20% to allow for the adjustment of factors (e.g., withdrawals, missing data, and lost to follow-up). The questionnaire survey and tap water sampling were conducted only in households who drank their water from the tap, covering different sociodemographic characteristics (gender, age, marital status, education level, employment status, household income, and household size) and housing types (landed and high-rise).

**Figure 6:**
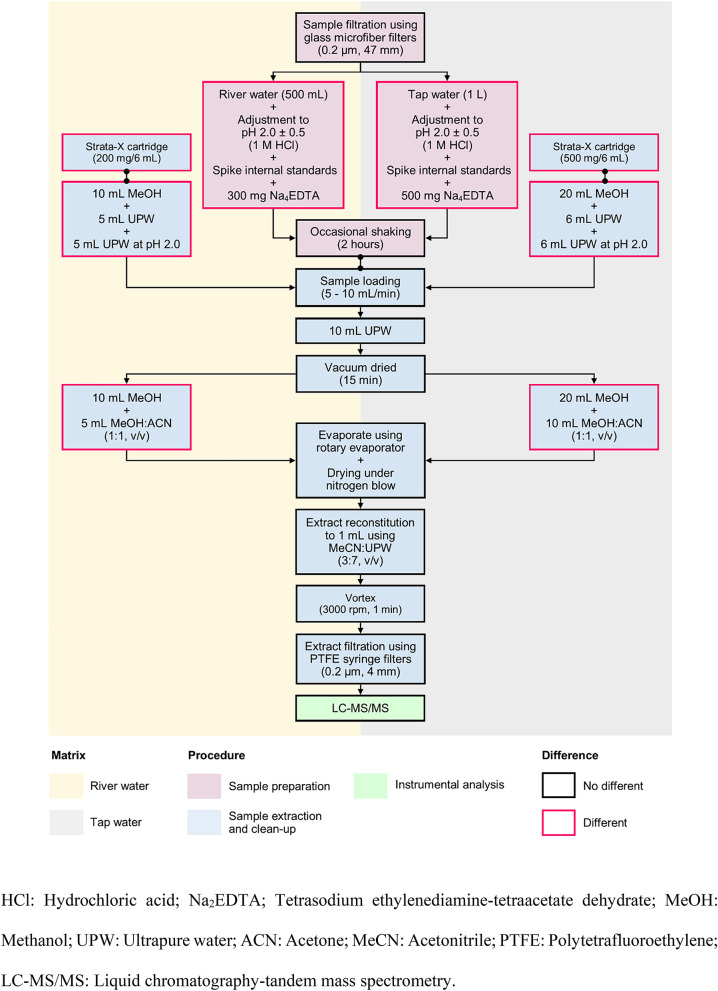
Analytical procedure for the analysis of multiclass EDCs in (**a**) river water and (**b**) tap water.

Household tap water sampling (1 L; *n* = 155) at Putrajaya residential areas (Fig. 5a) occurred between July and October 2018. Water samples were directly collected from running taps after two minutes of flushing. Surface river water (500 mL; *n* = 10) was also sampled along the Langat River, a main water source for nearby the drinking water treatment plant, thus covering upstream, before and after drinking water treatment plant, to downstream (Fig. 5a). A portable YSI Pro Plus multiparameter meter (YSI Inc., Yellow Springs, Ohio, USA) and Hach 2100P portable turbidimeter (HACH Lange GmbH, Dusseldorf, Germany) were used on-site for the measurement of physicochemical properties of water samples, including temperature, pH, dissolved oxygen, conductivity, salinity, oxidation–reduction potential, total dissolved solids, and turbidity, testing the ambient water quality. Using precleaned methanol-rinsed amber glass bottles, water samples were collected and transported in an icebox (± 4 °C) for the following laboratory analysis process. Water samples were filtered using glass microfiber filters (Whatman, Buckinghamshire, UK), fortified with excess ascorbic acid (Sigma-Aldrich Chemie GmbH, Steinheim, Germany), and extracted within 48 h of collection. Samples were pretreated, spiked with isotope-labeled surrogate standards, and added with tetrasodium ethylenediamine-tetraacetate dehydrate (Na_2_EDTA; Merck, Darmstadt, Germany) prior to solid phase extraction, as shown in Fig. 6. The extraction process of river and tap water samples was adopted from Wee et al*.*^20,56^, respectively. Then, target EDCs were quantified using liquid chromatography–tandem mass spectrometry (LCMS-8030 Tandem Quadrupole Mass Spectrometer, Shimadzu, Japan), employing both positive and negative electrospray ionization^20^.

### Quality assurance and quality control

High purity stock standards for the target EDCs, i.e., dexamethasone (99.2%), primidone (99.5%), propranolol (99.3%), ciprofloxacin (94%), caffeine (99.9%), sulfamethoxazole (99%), diclofenac (99.5%), chloramphenicol (99.3%), triclosan (99.5%), testosterone (99%), progesterone (99.3%), estrone (99.5%), 17β-estradiol (96.6%), 17α-ethynylestradiol (98%), bisphenol A (98.5%), 4-octylphenol (99.7%), 4-nonylphenol (99.3%), and diazinon (98.9%) were supplied by Dr. Ehrenstorfer GmbH (Augsburg, Germany). Methanol (LC–MS and HPLC grade), acetonitrile (LC–MS and HPLC grade), and acetone (HPLC grade) for the analytical protocol were sourced from Thermo Fisher Scientific (New Jersey, USA). Ultrapure water (resistivity of 18.2 MΩ cm at 25 °C) was acquired from a water purification system (Millipore, Massachusetts, USA). Briefly, the method of recovery of the target EDCs in tap water and river water analysis was validated at a spiking concentration of 100 ng/L, ranging from 55.97 to 146.42% and 25.53 to 156.56%, respectively. Notably, 4-nonylphenol was excluded in the river water analysis due to the limitations of the extraction method and matrix interference. The method detection limit (tap water: 0.01–2.56 ng/L; river water: 0.01–0.45 ng/L) and method quantification limit (tap water: 0.04–8.55 ng/L; river water: 0.02–1.50 ng/L) were tested using S/N ratios of 3:1 and 10:1, accordingly. Good linearity of the target compounds with correlation coefficients ranged above 0.9. Method precision (repeatability and reproducibility) was validated, where relative standard deviation values were observed below 15% following intra-day and inter-day analyses of a standard mixture (100 µg/L). Ionization suppression of the targeted compounds with matrix effect values of less than 100% was subsequently controlled using isotopically labeled standards primidone (D5; 98%), sulfamethoxazole (D4; 98%), diclofenac (D4; 98%), diazinon (D10; 98%), 17β-estradiol (D4; 95–97%), 17α-ethynylestradiol (D4; 97–98%), and bisphenol A (D8; 98%) (Toronto Research Chemicals, Toronto, Canada). The method validation process for river and tap water analyses is summarized in Supplementary Table S1.

### Human health risk assessment

The EDI, derived from the maximum detected concentration of EDCs (C_s_) and the daily water intake per body weight (DWI/BW), suggests the daily exposure to EDCs in drinking water. Human health risks for detected EDCs were appraised using the RQ approach, where the RQ of each detected EDC for the respective age group was estimated using Eq. (1), dividing the C_s_ by the respective drinking water equivalent level (DWEL).

1 $${\text{RQ }} = {{{\text{ C}}_{{\text{s}}}}/{{\text{DWEL}}}}$$

2 $${\text{DWEL }} = \, \left( {{\text{ADI }} \times {\text{ BW }} \times {\text{ HQ}}} \right)/\left( {{\text{DWI }} \times {\text{ AB }} \times {\text{ FOE}}} \right)$$

DWEL, as demonstrated in Eq. (2), is dependent on age group and consumption. Thus, the frequency of exposure (FOE), body weight (BW), and daily drinking water intake (DWI) were adopted based on a questionnaire survey for more deterministic local levels of exposure to EDCs. This occurred is because human growth varies across countries^57^. The questionnaire was validated by experts and pretested before the actual study was conducted (Cronbach's alpha value = 0.935). The questionnaire was copyrighted (LY2018000940) by Putra Science Park and ethically approved (JKEUPM-2017-181) by the Ethics Committee for Research Involving Human Subjects of Universiti Putra Malaysia. All the methods and materials were carried out in accordance with relevant guidelines and regulations. The questionnaires were hand-delivered or distributed via online platforms to Putrajaya residents. The present study involved only residents who were at least 18 years old. Respondents were informed about the nature and the purpose of the study and given a choice to participate, made by favoring or opposing answering the questionnaire. Data of children aged younger than 18 years were collected from the respective parents or guardians upon the adults’ agreement.

The hazard quotient (HQ) and gastrointestinal absorption rate (AB) were assumed to be 1^6^. The ADIs of detected EDCs were established based on previous studies^6,30–35^. Exposure factor definitions and values for human health risk assessment are tabulated in Table 2. An RQ of greater than 1 suggests the likely presence of a risk to human health among the exposed individuals and populations.

**Table 2:** Exposure factor definitions and values for human health risk assessment.

Parameter	Definition	Unit	Value	References
C_s_	Maximum detected concentration of EDCs	ng/L	Monitoring data	This study
ADI	Acceptable daily intake	ng/kg/day	15–150,000	^6,30–35^
BW	Body weight	kg	Questionnaire data	This study
	- Adults			
	- Children			
HQ	Hazard quotient	NA	1	^6^
DWI	Daily water intake	L/day	Questionnaire data	This study
AB	Gastrointestinal absorption rate	NA	1	^6^
FOE	Frequency of exposure	NA	Questionnaire data	This study

*NA* not applicable.

### Data analysis

The variation and distribution of EDCs in the drinking water supply system were evaluated through descriptive statistics. An independent *t* test was used to determine statistically significant differences between the physicochemical properties and concentrations of EDCs in drinking water source and supply samples. The analysis of data was accomplished using statistical software program version 22 (IBM Corporation, Armonk, New York, USA).

## Supplementary information

Supplementary Tables

## Data Availability

Data are available on request by contacting the corresponding author (zaharin@upm.edu.my).
