# Supplementary material for: Occurrence of multiclass endocrine disrupting compounds in a drinking water supply system and associated risks
Source: Sci Rep. 2020 Oct 20;10:17755. doi: 10.1038/s41598-020-74061-5 (PMC7576597; doi:10.1038/s41598-020-74061-5)
Supplement: Supplementary file 1 — Supplementary Tables [file 41598_2020_74061_MOESM1_ESM.docx]

SUPPLEMENTARY INFORMATION

**Occurrence of multiclass endocrine disrupting compounds in a drinking water supply system and associated risks**

Sze Yee Wee^a^, *Ahmad Zaharin Aris^a,d^, Fatimah Md. Yusoff^b,d^, Sarva Mangala Praveena^c,d^

*^a^**Department of Environment, Faculty of Forestry and Environment, Universiti Putra Malaysia, 43400 UPM Serdang, Selangor, Malaysia.*

*^b^Department of Aquaculture, Faculty of Agriculture, Universiti Putra Malaysia, 43400 UPM Serdang, Selangor, Malaysia.*

*^c^Department of Environmental and Occupational Health, Faculty of Medicine and Health Sciences, Universiti Putra Malaysia, 43400 UPM Serdang, Selangor, Malaysia.*

*^d^International Institute of Aquaculture and Aquatic Sciences, Universiti Putra Malaysia, 71050 Port Dickson, Negeri Sembilan, Malaysia*

*Corresponding author Ahmad Zaharin Aris

Tel: +603-97697455

Fax: +603-89438109

E-mail: zaharin@upm.edu.my

ORCID: 0000-0002-4827-0750

**Supplementary Table S1** QA/QC parameters for the analysis of target EDCs in tap and river water.

| EDC group | Compound | QA/QC parameter | | | | | | | | | | |
| --- | --- | --- | --- | --- | --- | --- | --- | --- | --- | --- | --- | --- |
|  |  | Recovery ± RSD (%) | | MDL (ng/L) | | MQL (ng/L) | | Matrix effect (%) | | *r* value | Intra-day precision (% RSD) | Inter-day precision (% RSD) |
|  |  | T | R | T | R | T | R | T | R |  |  |  |
| Hormone | Testosterone | 88.36 ± 24.47 | 105.86 ± 0.44 | 0.06 | 0.02 | 0.20 | 0.07 | -19.36 | -67.47 | 0.9944 | 5.54 | 2.08 |
|  | Progesterone | 109.94 ± 29.30 | 117.58 ± 1.96 | 0.04 | 0.02 | 0.14 | 0.07 | 4.15 | -69.85 | 0.9974 | 13.30 | 6.02 |
|  | Estrone | 101.12 ± 8.51 | 97.60 ± 0.90 | 0.08 | 0.02 | 0.26 | 0.08 | 24.0 | -78.81 | 0.9879 | 0.11 | 0.60 |
|  | 17β-estradiol | 96.71 ± 9.83 | 106.42 ± 6.36 | 0.58 | 0.17 | 1.92 | 0.56 | 11.5 | -86.60 | 0.9876 | 0.84 | 1.61 |
|  | 17α-ethynylestradiol | 103.05 ± 2.13 | 67.11 ± 2.07 | 0.51 | 0.35 | 1.69 | 1.15 | 22.4 | -83.36 | 0.9990 | 9.65 | 5.54 |
| Pharmaceutical | Dexamethasone | 106.92 ± 37.53 | 86.12 ± 6.11 | 0.36 | 0.10 | 1.20 | 0.33 | -3.34 | -33.75 | 0.9841 | 4.44 | 0.76 |
|  | Primidone | 91.93 ± 55.09 | 99.26 ± 2.95 | 0.14 | 0.08 | 0.48 | 0.27 | -23.45 | -69.30 | 0.9996 | 0.50 | 1.65 |
|  | Propranolol | 90.07 ± 44.94 | 156.56 ± 4.88 | 0.03 | 0.01 | 0.09 | 0.03 | 14.47 | -57.99 | 0.9969 | 0.33 | 5.95 |
|  | Ciprofloxacin | 146.42 ± 14.63 | 62.66 ± 53.83 | 0.05 | 0.05 | 0.18 | 0.18 | -69.07 | -83.16 | 0.9014 | 5.14 | 3.34 |
|  | Caffeine | 133.67 ± 52.26 | 128.92 ± 8.03 | 0.01 | 0.01 | 0.04 | 0.05 | -13.56 | -53.21 | 0.9978 | 6.47 | 3.72 |
|  | Sulfamethoxazole | 107.08 ± 12.84 | 97.54 ± 2.80 | 0.03 | 0.01 | 0.11 | 0.02 | -17.59 | -33.58 | 0.9995 | 1.49 | 4.81 |
|  | Diclofenac | 95.27 ± 34.59 | 109.99 ± 3.56 | 2.56 | 0.45 | 8.55 | 1.50 | -46.93 | -72.41 | 0.9300 | 14.37 | 0.86 |
|  | Chloramphenicol | 85.00 ± 30.41 | 55.86 ± 2.97 | 0.23 | 0.09 | 0.75 | 0.30 | -62.55 | -82.54 | 0.9985 | 8.98 | 11.55 |
|  | Triclosan | 86.01 ± 43.21 | 75.32 ± 1.60 | 1.29 | 0.17 | 4.29 | 0.56 | -49.68 | -89.03 | 0.8909 | 2.81 | 2.49 |
| Plasticizer | Bisphenol A | 119.02 ± 10.03 | 111.37 ± 3.46 | 0.04 | 0.03 | 0.12 | 0.10 | 39.3 | -79.16 | 0.9953 | 5.86 | 1.09 |
|  | 4-octylphenol | 68.57 ± 54.83 | 25.53 ± 6.45 | 0.25 | 0.21 | 0.83 | 0.70 | 53.3 | -93.84 | 0.9812 | 5.62 | 4.16 |
|  | 4-nonylphenol | 55.97 ± 33.97 | NA | 2.29 | NA | 7.23 | NA | 37.6 | NA | 0.9946 | 3.41 | 2.05 |
| Pesticide | Diazinon | 100.95 ± 26.41 | 109.81 ± 4.76 | 0.08 | 0.01 | 0.26 | 0.04 | -15.86 | -50.20 | 0.9999 | 3.29 | 0.63 |
| Minimum |  | 55.97 | 25.53 | 0.01 | 0.01 | 0.04 | 0.02 | -69.07 | -93.84 | 0.8909 | 0.11 | 0.60 |
| Maximum |  | 146.42 | 156.56 | 2.56 | 0.45 | 8.55 | 1.50 | 53.3 | -33.58 | 0.9999 | 14.37 | 11.55 |

MDL: Method detection limit; MQL: Method quantification limit; NA: Not available; T: Tap water; R: River water.
